# Ambient Light Regulates Retinal Dopamine Signaling and Myopia Susceptibility

**DOI:** 10.1167/iovs.62.1.28

**Published:** 2021-01-27

**Authors:** Erica G. Landis, Han Na Park, Micah Chrenek, Li He, Curran Sidhu, Ranjay Chakraborty, Ryan Strickland, P. Michael Iuvone, Machelle T. Pardue

**Affiliations:** 1 Department of Neuroscience, Emory University, Atlanta, Georgia, United States; 2 Department of Ophthalmology, Emory University, Atlanta, Georgia, United States; 3 Department of Pharmacology, Emory University, Atlanta, Georgia, United States; 4 Center for Visual and Neurocognitive Rehabilitation, Atlanta VA Healthcare System, Atlanta, Georgia, United States; 5 Biomedical Engineering, Georgia Institute of Technology and Emory University, Atlanta, Georgia, United States

**Keywords:** myopia, scotopic, photopic, mesopic, lens defocus

## Abstract

**Purpose:**

Exposure to high-intensity or outdoor lighting has been shown to decrease the severity of myopia in both human epidemiological studies and animal models. Currently, it is not fully understood how light interacts with visual signaling to impact myopia. Previous work performed in the mouse retina has demonstrated that functional rod photoreceptors are needed to develop experimentally-induced myopia, alluding to an essential role for rod signaling in refractive development.

**Methods:**

To determine whether dim rod-dominated illuminance levels influence myopia susceptibility, we housed male C57BL/6J mice under 12:12 light/dark cycles with scotopic (1.6 × 10^−3^ candela/m^2^), mesopic (1.6 × 10^1^ cd/m^2^), or photopic (4.7 × 10^3^ cd/m^2^) lighting from post-natal day 23 (P23) to P38. Half the mice received monocular exposure to −10 diopter (D) lens defocus from P28–38. Molecular assays to measure expression and content of DA-related genes and protein were conducted to determine how illuminance and lens defocus alter dopamine (DA) synthesis, storage, uptake, and degradation and affect myopia susceptibility in mice.

**Results:**

We found that mice exposed to either scotopic or photopic lighting developed significantly less severe myopic refractive shifts (lens treated eye minus contralateral eye; –1.62 ± 0.37D and −1.74 ± 0.44D, respectively) than mice exposed to mesopic lighting (–3.61 ± 0.50D; *P* < 0.005). The 3,4-dihydroxyphenylacetic acid /DA ratio, indicating DA activity, was highest under photopic light regardless of lens defocus treatment (controls: 0.09 ± 0.011 pg/mg, lens defocus: 0.08 ± 0.008 pg/mg).

**Conclusions:**

Lens defocus interacted with ambient conditions to differentially alter myopia susceptibility and DA-related genes and proteins. Collectively, these results show that scotopic and photopic lighting protect against lens-induced myopia, potentially indicating that a broad range of light levels are important in refractive development.

Myopia is increasing at epidemic rates in many countries; in the United States its prevalence has reached an alarming 42% in the last three decades.^1^ Although genetic factors are known to contribute to myopia development,^2^ the magnitude of this increased prevalence suggests that environmental factors significantly contribute to myopia development. To find preventative strategies to curb this increase in myopia prevalence, the field has focused on environmental light exposure that may be driving myopia development and progression.^3^^,^^4^

It has been shown that children who spend more time outdoors in bright light are less likely to become myopic or to experience a progression of their myopia.^5^^–^^8^ Recently, clinical trials have shown the effectiveness of reducing myopia with intentional increases in time outdoors in children.^6^^,^^7^^,^^9^^,^^10^ Several studies using animal models such as tree shrews, chicks, and macaques have confirmed the protective effect of bright ambient illuminance on myopia development using controlled laboratory conditions.^11^^–^^15^ However, some studies have not reported benefits from bright light, including in monkeys with lens-induced myopia (LIM) and form-deprived chickens exposed to outdoor, bright light.^14^^,^^15^ Thus it is unknown whether bright light is the optimal or only potential environmental light that is beneficial.

Our visual system is optimized to function over a broad range of light conditions. The retina can detect a single photon of light through rod pathways and function under 10^8^ to 10^15^ photons/cm^2^/s of light using cone- and melanopsin-mediated pathways.^16^^,^^17^ It has been assumed that refractive development is driven by cone pathways, which are responsible for high acuity vision and perception in bright light. However, there is a growing body of data that support rod pathway contributions to visually-driven eye growth.^18^^,^^19^ Rod photoreceptor dysfunction in the *Gnat1−*/− mouse resulted in abnormal refractive development and the inability to respond to form deprivation myopia (FDM).^20^ Furthermore, nonmyopic children have been shown to spend more time in either dim or bright light compared to myopic age-matched peers.^21^ Thus both rod and cone pathways stimulated by dim and bright light, respectively, may be required for optimal eye growth.^22^

The exact mechanisms underlying refractive development and myopia remain elusive. However, retinal dopamine (DA) has been implicated as a stop signal for myopic eye growth because it is decreased with FDM^23^^,^^24^ or LIM.^25^^–^^28^ DA has also been implicated as the signaling mechanism in the protective effects of bright light.^12^^,^^29^ Retinal DA synthesis^30^ and quantified DA release^31^ have been shown to increase with light exposure. However, the exact mechanism by which bright light increases retinal DA levels or DA activity is not clear. DA signaling in the retina is highly compensatory, and therefore several DA-related proteins could contribute to increased DA signaling to prevent myopia.^32^ Other retinal proteins that control the presence and localization of DA include vesicular monoamine transporter 2 (VMAT2) to package DA into vesicles for storage and release,^33^ dopamine transporter (DAT) to move released DA back into the cell and clear DA from the extracellular space,^32^ and monoamine oxidase A and B (MAO) to degrade DA after uptake (Fig. 5A).

In this study we used the mouse model of myopia, leveraging a well-characterized mammalian retina that responds to FDM and lens defocus.^34^^–^^39^ We examined the effect of lens defocus in mice housed in three different environmental illuminance levels: scotopic, mesopic, and photopic lighting. To fully analyze the role of DA signaling, we evaluated various DA-related proteins with long duration (2 weeks) light exposures, as well as short duration (3 hours) light exposures to test acute changes in retinal DA dynamics. We hypothesized that both dim and bright light exposure would reduce lens defocus myopia through DA-mediated mechanisms.

## Methods

### Animals and Experimental Design

**Figure 1.**
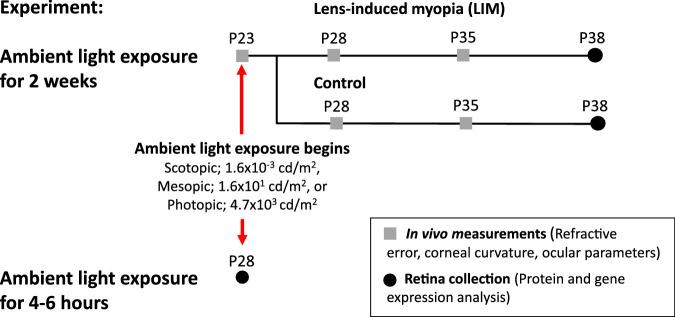
Experimental Design of the Study.

Male wild-type C57BL/6J mice (Jackson Labs, Bar Harbor, ME, USA) were used at postnatal day 23 (P23). Mice were placed into scotopic (1.6 × 10^−3^ cd/m^2^, *n* = 101), mesopic (1.6 × 10^1^ cd/m^2^, n = 100), or photopic (4.7 × 10^3^ cd/m^2^, *n* = 101) lighting conditions on a 12:12-hour light/dark (LD) cycle (Fig. 1).

Animals were housed individually at the Atlanta Veterans Affairs Medical Center with mouse chow and water available as desired. Cages were topped with wire lids, and food was provided at the bottom of the cage to prevent shadows. Mice were monitored daily during the experiment. All procedures were approved by the Atlanta VA Institutional Animal Care and Use Committee and adhered to the ARVO Statement for the Use of Animals in Ophthalmic and Vision Research.

### Light Exposure

Mice were exposed to one of three lighting conditions using a custom-made light-tight box (Actimetrics, Wilmette, IL, USA) which protected the animals from light pollution present in the room. Experimental illuminance levels stimulated different photoreceptors, including rod-only scotopic (1.6 × 10^−3^ cd/m^2^), mixed rod and cone mesopic (1.6 × 10^1^ cd/m^2^), and cone-dominated and melanopsin-stimulating photopic (4.7 × 10^3^ cd/m^2^). The ceiling of the box contained a series of white LEDs that were covered with neutral density filters to obtain the desired candela/m^2^ values for scotopic illuminance. Photopic lighting was created with white LEDs in a commercial panel (Fancier Studio, Hayward, CA, USA) placed four to six inches from the top of the cages. Ambient light levels were measured at the floor of the cage using a light meter (VWR Traceable Dual-Range, Radnor, PA, USA) and spectrophotometer (BWTek Exemplar Spectrophotometer, Newark, DE, USA). Ambient temperatures in the light environments were within the normal range for the animal facility (22°C–24°C). Animals were kept in their prescribed light treatments for the entire course of the experiment except when measurements were being done, during which cages were protected from light exposure or kept in the dark.

To evaluate DA dynamics after acute exposure to the light, a subset of P28 mice were placed in either scotopic (*n* = 12), mesopic (*n* = 15), or photopic light (*n* = 14) and sacrificed after three hours of the light phase (CT 3) on the first day of exposure (Fig. 1). Retinas were collected under the assigned illuminance level for each mouse and immediately frozen on dry ice for protein and gene expression analysis.

### Refractive and Ocular Measurements

Refractive and ocular measurements were performed at P23, 28, and 35 as previously described (Fig. 1).^32^^,^^40^^,^^41^ Pupils were dilated with 1% tropicamide (Bausch + Lomb, Bridgewater, NJ, USA). Refractive errors were measured with a custom-made photorefractor^34^^,^^42^ in both awake and anesthetized conditions (ketamine [80 mg/kg]/xylazine [16 mg/kg]). Mice that had a difference in refractive error between the two eyes that was greater than 2.5D at baseline was considered amblyopic and was excluded from the study (*n* = 3). Corneal curvature was measured with a custom-made keratometer.^42^^,^^43^ Cross-sectional images of the mouse eye were obtained using a 1310-mm spectral domain optical coherence tomography system (Bioptigen Inc., Durham, NC, USA). These images were then used to measure axial length, anterior and vitreous chamber depth, and corneal, lenticular, and retinal thickness.^44^ After testing, mice were given yohimbine (2.1 mg/kg) to reverse the effects of xylazine and prevent corneal ulcers^45^ and were then allowed to recover. Some optical coherence tomography images were of low quality and were not included in the analysis.

### LIM

Animals were anesthetized (ketamine [80 mg/kg]/xylazine [16 mg/kg]), and a head-mounted lens holder was surgically attached, as previously described.^34^ A clear −10 D lens (12 mm diameter, 6.75 base curve, 0.2 mm thickness, X-Cel Specialty Contacts, Duluth, GA, USA) was held with a custom-made frame over the right eye (OD) of a subset of animals from each light level (scotopic: *n* = 21; mesopic: *n* = 20; photopic: *n* = 20 treated with LIM). Frames were “threaded” through a surgically implanted head pedestal and held in place with the tightening of an aluminum cube around the frame. The cube also held a “balance bar” that rested on the left side of the face.^34^^,^^42^ Defocus lenses were kept in place until the end of the experiment, checked daily for fit and compliance, and cleaned as needed. The untreated left eye served as a paired control, referred to here as the contralateral eye.

### Retina Collection for High-Performance Liquid Chromatography Detection of Dopamine

For the long duration experiment, animals were sacrificed at P38. Eyes were enucleated, and retinas were collected four to six hours after light onset (control: *n* = 15/light level; LIM: *n* = 16/light level). Retinas were immediately frozen on dry ice and kept in storage at −80°C.

Collected retinas from both eyes of LIM and control mice were analyzed by high-performance liquid chromatography (HPLC) to determine levels of DA and its metabolite 3,4-dihydroxyphenylacetic acid (DOPAC).^46^^,^^47^ Briefly, retinas were homogenized in 0.1 N perchloric acid, 0.01% sodium metabisulfite with 25 ng/mL 3,4-dihydroxybenzylamine, and spun in a centrifuge. Supernatant was injected into a Beckman Ultrasphere 5 µm ODS column, 250 × 4.6 mm (Fullerton, CA, USA). The mobile phase consisted of 0.1M phosphoric acid, 0.1 mM EDTA, 0.35 mM sodium octyl sulfate and 6% acetonitrile at pH 2.7. Analyzed peaks were identified by retention time and compared to those of external standards quantified by peak area.

Retinas of mice given lens defocus were tested as individual samples (lens defocus and contralateral) whereas the right and left eyes of control mice were pooled for analysis. Samples were normalized to total retinal protein content determined by Lowry Assay.

### Gene Expression of Dopamine-Related Proteins

To measure the gene expression of DA-related proteins (TH: *Th*, VMAT2: *Slc18a2*, DAT: *Slc6a3*, MAO-A: *Maoa*, and MAO-B: *Maob*), retinas frozen in Ribolock (ThermoFisher, Waltham, MA, USA) from control (*n* = 6/light level) and LIM treated (*n* = 8/light level) mice from each ambient light level were homogenized in RLT buffer (Qiagen, Venlo, Netherlands) using a Tissuelyser LT (Qiagen). RNA was extracted using the Qiagen RNAeasy Qiacube Kit (Qiagen). A QuantiNova cDNA synthesis kit (Qiagen) was used to make cDNA as per the manufacturer's protocol.

Digital droplet polymerase chain reaction (ddPCR) was used to determine relative quantities of transcripts for the genes of interest. Fluorescein amidite (FAM)–labeled hydrolysis probe assays for *Th*, *Slc18a2*, *Slc6a3*, *Maoa*, and *Maob*, and a hexachloro-fluorescein (HEX)–labeled probes assay for hypoxanthine phosphoribosyltransferase (HPRT) (Table 1). Data were analyzed using QuantiSoft analysis software (Bio-Rad), which uses a Poisson distribution model to calculate the number of starting target template molecules in each well from the number of FAM- and HEX-positive droplets.

**Table 1.** Probes Used in ddPCR Analysis of Genes Related to DA Dynamics

Gene, Protein	Hydrolysis Fluor	Company	Catalog Number
HPRT	HEX	IDT	Mm.PT.39a322214828
*Th*, TH	FAM	Bio-Rad	dMmuCPE5121062
*Slc18a2*, VMAT2	FAM	IDT	Mm.PT.58.42226157
*Slc6a3*, DAT	FAM	IDT	Mm.PT.58.12888045
*Maoa*, MAOA	FAM	IDT	Mm.PT.58.8802827
*Maob*, MAOB	FAM	IDT	Mm.PT.58.33530177

### Western Blot Detection of Dopamine-Related Proteins

Western blots were performed to measure levels of DA related proteins TH, pTH^Ser40^ (tyrosine hydroxylase phosphorylated at amino acid site Serine-40), and VMAT2. Retinas (control: *n* = 6–7/light level; LIM: *n* = 7/light level) collected between four to six hours after light onset were frozen, then homogenized in RIPA buffer (Teknova, Hollister, CA, USA) with protease inhibitors (Roche, Penzberg, Germany). Only data from control OD and lens defocus OD retinas were used. A bicinchoninic acid assay was used to normalize protein concentrations between samples.

From each sample 15 µg protein was run on a precast gel and transferred to polyvinylidene fluoride (PVDF) membranes (Bio-Rad). Membranes were treated with primary antibodies (Table 2) in PBS-T with 5% BSA at 4°C overnight and then stained with secondary antibodies and HRP conjugate (horseradish peroxidase, Bio-Rad) in PBS-T with 5% BSA at room temperature for one hour before imaging in a Bio-Rad ChemiDoc MP Imaging System. To quantify protein bands, the Bio-Rad computer software was used to detect band intensity and normalize to total protein in each well. Intensity values for each sample were then normalized to those of a mesopic-housed control mouse, which were run on every blot. The α-tubulin was used as a loading control.

**Table 2.** Antibodies Used to Measure DA-Related Proteins in Retinal Tissue

Protein Target	Source	Concentration	Secondary Antibody
TH	EMD Millipore (AB152)	1:1,000	Pierce Goat AntiRabbit IgG (31460, 1:5,000)
pTH^Ser40^	Sigma Aldrich (T9573)	1:1,000	Pierce Goat AntiRabbit IgG (31460, 1:5,000)
VMAT2	Custom-made^*^	1:1,000	Pierce Goat AntiRabbit IgG (31460, 1:5,000)
α-Tubulin	Abcam (ab4074)	1:5,000	EMD Millipore Goat AntiRabbit IgG (AP132, 1:10,000)

* Custom-made VMAT2 antibody.^112^

### Statistical Analysis

Refractive error and other ocular parameters of experimental and control groups were compared across light levels by comparing means for control mice (average of both eyes), lens treated eyes, and contralateral eyes using one-way and two-way ANOVA (GraphPad Prism 8, San Diego, CA, USA; and SigmaStat, San Jose, CA, USA), as detailed below. The mean myopic shift (lens defocus–treated eye minus contralateral eye) for lens defocus treated mice in each light level was used as a measure of intra-animal effect. For comparisons of refractive error, corneal curvature, and ocular axial parameters, two-way repeated measures ANOVAs were used to examine the effects of light treatment (scotopic, mesopic, photopic) and time (23, 28, and 36 days) or lens defocus treatment (control, contralateral left, and lens-treated right eyes) and time (23, 28, and 36 days). When interaction effects were significant, Holm Sidak post hoc comparisons were reported. One-way ANOVAs were used to compare groups at the final timepoint. Additionally, we performed Pearson correlation analysis of refractive error and axial length on a subset of mice in the control and lens-treated eye from each light treatment, including data from both the 28- and 36-day timepoint (GraphPad Prism 8). This analysis provided information about the trends in axial elongation versus refractive error in control and lens-treated mice.

Results of experiments done to measure DA and DOPAC, gene expression levels, and DA related protein levels were analyzed using two-way ANOVAs (GraphPad Prism) and Holm Sidak post hoc comparisons. No differences were found between contralateral and lens treated eyes, therefore only lens treated data is presented here. Acute light exposure results were analyzed with one-way ANOVAs with Holm Sidak post hoc comparisons. Representative average running distances based on running wheels (Wheel Analysis Software; Med Associates, Inc, Fairfax, VT, USA) placed in cages of singly housed animals from ZT 1 and ZT 13 (zeitgeber time, beginning at light onset) were compared across light levels using a two-way repeated measures ANOVA with Holm Sidak post hoc comparisons. For all analyses, significance was set at alpha of 0.05. Data shown here are means ± SEM.

## Results

### Scotopic and Photopic Lighting Decreased Myopic Shift to Lens Defocus

**Figure 2.**
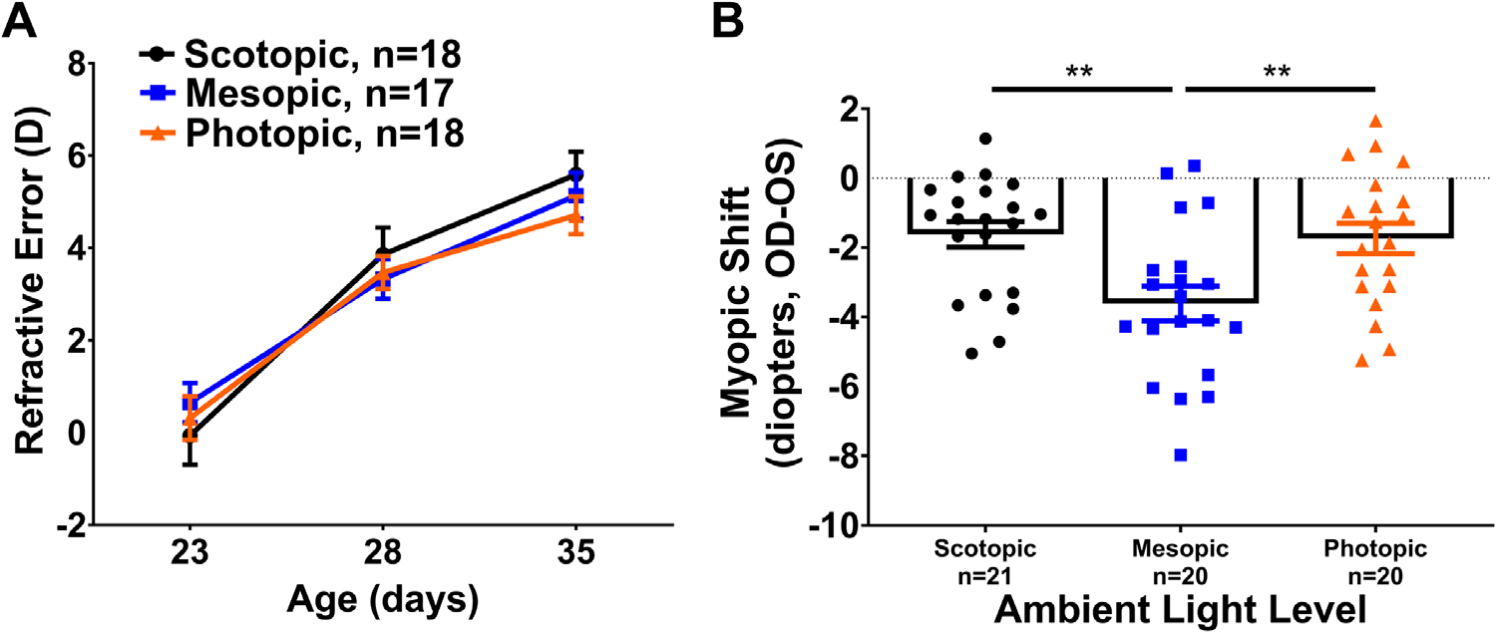
Scotopic and photopic light exposure inhibited lens induced myopia in mice. (**A**) Control mice housed in scotopic (*black*), mesopic (*blue*), or photopic (*orange*) light showed identical refractive error development. (**B**) The myopic shift at P35 of lens treated mice exposed to mesopic light is greater than mice exposed to scotopic or photopic light (one-way ANOVA F(2,58) = 6.50; *P* < 0.01). All data shown are mean ± SEM, *n* = 17–21/lens defocus group, post hoc comparisons indicated by **P < 0.01.

Animals were housed in either scotopic, mesopic, or photopic light (defined in Fig. 1) during the light phase of a 12:12-hour light/dark cycle beginning at P23. At P28, a subset of mice was treated with monocular defocus using −10D lenses. Control mice without lens defocus showed no significant differences in refractive development between light exposure groups from P23 to P35 (two-way repeated ANOVA, F(4, 100) = 1.26; *P* = 0.29; Fig. 2A).

To directly compare the effect of ambient light on the response to monocular lens defocus, the myopic shift (lens treated eye [OD] − contralateral eye [OS]) was calculated at P35 for each light level. Lens defocus treated mice exposed to mesopic light had significantly greater myopic shifts (−3.61 ± 0.50 D) than mice exposed to scotopic (−1.62 ± 0.37 D; *P* < 0.01) or photopic (−1.74 ± 0.44 D; *P* < 0.01) light (one-way ANOVA F(2,58) = 6.50; *P* < 0.01; Fig. 2B; absolute refractive values shown in Supplementary Fig. S1). Scotopic and photopic light exposure produced similar myopic shifts with no significant differences between groups. No significant differences were found between control and contralateral eyes under any light exposure group (Supplementary Fig. S1).

### Mesopic Lighting Differentially Altered Ocular Parameters

**Figure 3.**
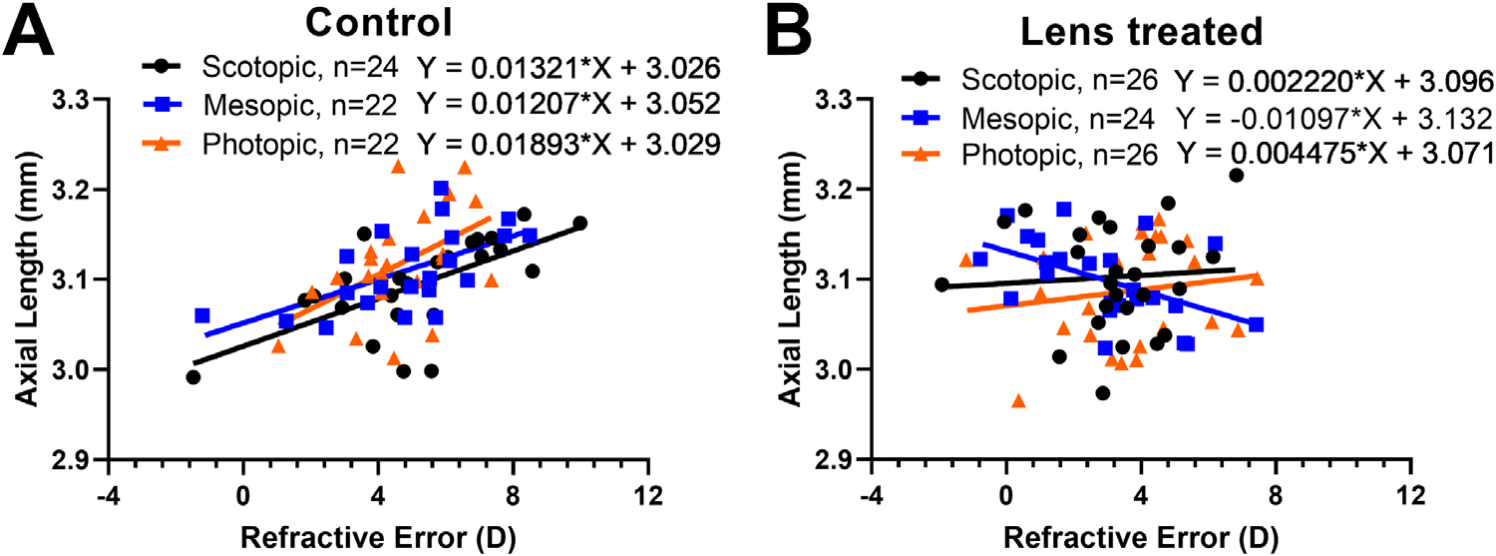
Axial length correlated with refractive error in control and mesopic housed lens defocus mice. (**A**) In control animals, axial length and refractive error at 28 and 35 days of age were positively correlated under scotopic (*black circles*, *R*^2^ = 0.407; *P* < 0.001), mesopic (*blue squares*, *R*^2^ = 0.368; *P* < 0.01) and photopic (*orange triangles*, *R*^2^ = 0.249; *P* < 0.05) light. (**B**) With lens defocus treatment, mice housed in either scotopic or photopic light showed a nonsignificant, positive correlation between axial length and refractive error at 28 and 35 days for lens-treated eyes. However, mesopic light exposure resulted in a negative correlation between refractive error and axial length (*R*^2^ = 0.261; *P* < 0.05), indicating more myopic refractive errors were associated with longer axial lengths. n = number of eyes used in the analysis at both timepoints; only right eyes are shown for both panels.

A correlation of refractive error and axial length indicated that animals treated with mesopic light tended to develop exaggerated myopic growth compared with scotopic and photopic light. The refractive errors of control eyes at 28 and 35 days were significantly and positively correlated to increased axial length across all of the ambient illuminance levels (scotopic: *R*^2^ = 0.407; *P* < 0.001; mesopic: *R*^2^ = 0.368; *P* < 0.01, photopic: *R*^2^ = 249; *P* < 0.05, Fig. 3A). In lens-treated eyes, mice housed in mesopic light had a significant, negative correlation between refractive error and axial length, indicating that eyes with lower refractive errors (greater relative myopia) tended to have larger axial lengths (*R*^2^ = 0.261; *P* < 0.05, Fig. 3B). Because the scotopic and photopic lighting groups had smaller myopic shifts, it appears that this resulted in less axial elongation, resulting in nonsignificant correlations when comparing refractive error and axial length (scotopic: *R*^2^ = 0.005; *P* = 0.73; photopic: *R*^2^ = 0.023; *P* = 0.45).

**Figure 4.**
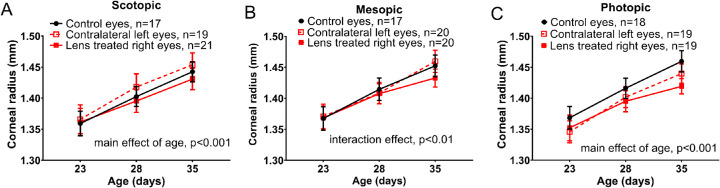
Lens treatment with mesopic light exposure steepened corneal curvature. (**A**) Corneal curvature of mice housed in scotopic light did not change with lens treatment but did increase with age (RM two-way ANOVA, main effect of age, F(2, 108) = 234.8; *P* < 0.001). (**B**) In mesopic light, the lens-treated eye had the lowest corneal curvature at P35 (RM two-way ANOVA, interaction effect, F(4, 108) = 3.7; *P* < 0.01). (**C**) Corneal curvature of mice housed in photopic light increased with age (RM two-way ANOVA, main effect of age, F(2, 106) = 195.6; *P* < 0.001) but did not change with lens treatment. Control eyes are shown in *solid black* lines, the naïve left eyes are shown in *red dashed* lines, and lens-treated eyes are shown in *solid red* lines. All data shown is mean ± SEM.

Furthermore, mice housed in mesopic lighting showed significantly steeper corneal curvature with age and lens defocus compared to the contralateral eye or age-matched controls (corneal radius of curvature at P35 lens-treated eye: 1.433 ± 0.014 mm; control eyes: 1.452 ± 0.018 mm; contralateral eyes: 1.460 ± 0.018 mm [RM two-way ANOVA, interaction effect, F(4,108) = 3.77; *P* < 0.01, Fig. 4B]). The corneal radius of curvature of eyes treated with lens defocus in scotopic (at P35 1.431 ± 0.0.017 mm) and photopic (at P35 1.425 ± 0.013 mm) illuminances were not significantly different than those of their illuminance-matched controls (at P35 scotopic: 1.450 ± 0.017 mm, photopic: 1.448 ± 0.017 mm, Figs. 4A, 4C).

Other ocular parameters measured included retinal thickness, lens thickness, corneal thickness, vitreous chamber depth, and anterior chamber depth. Although all measures showed an effect of age, no significant differences were found across treatment groups among any of these measures (Supplementary Fig. S2).

### Retinal Dopamine Levels and Metabolism Increased With Bright Light

**Figure 5.**
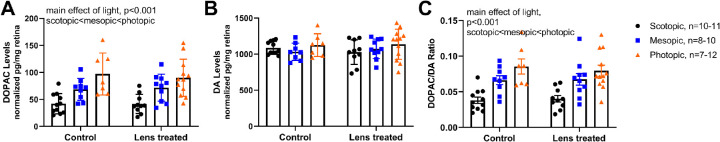
DA turnover increases with illuminance level. Retinas were collected four to six hours after light onset under each housing illumination level. (**A**) After ∼2 weeks of ambient light exposure, DOPAC levels were increased with higher light intensities in both treatment groups (two-way ANOVA, main effect of light, F(2,53) = 18.47; *P* < 0.001). For both treatment groups, all light level groups had significantly different DOPAC levels (*P* < 0.01). (**B**) None of the treatment groups showed changes in DA levels with light or lens defocus. (**C**) DOPAC/DA ratio increased with light indicating higher dopamine metabolism at higher light intensities (two-way ANOVA, main effect of light, F(2,53) = 19.45; *P* < 0.001). For both treatment groups, all light level groups had significantly different DOPAC/DA ratios (*P* < 0.05). Scotopic samples are represented by *black circles*, mesopic samples by *blue squares*, and photopic samples by *orange triangles*. Bars represent mean ± SEM.

To evaluate the potential role of DA in modulating refractive eye growth in the three light levels, retinal DA, DOPAC, and the DOPAC/DA ratio (indicative of DA turnover) were measured using HPLC. Within each light level, the treatment groups (control mice and lens defocus treated eyes) did not have significantly different levels of DOPAC or DA or DOPAC/DA ratios. However, as previously reported, DOPAC levels increased with increasing light levels^48^ (Two-way ANOVA [factors: light, treatment], main effect of light; F(2,53) = 18.47; *P* < 0.001; Fig. 5A). DOPAC levels were significantly different among all light level groups (*P* < 0.01); for both control and lens-treated eyes combined, scotopic-exposed mice had the lowest levels of DOPAC (41.83 ± 0.26 pg/mg), with increased levels in mesopic- (70.12 ± 1.60 pg/mg) and photopic-exposed mice (93.64 ± 0.3.60 pg/mg). DA levels were constant across both light levels and lens treatment (Fig. 5B). DOPAC/DA ratios showed the same increase across light levels as DOPAC levels (two-way ANOVA [factors: light, treatment], main effect of light, F(2,53) = 19.45; *P* < 0.001, Fig. 5C). DOPAC/DA ratios were significantly different among all light level groups (*P* < 0.05) with DOPAC/DA ratios for both control and lens-treated eyes combined significantly higher in photopic-exposed mice (0.04 ± 0.001) than mesopic-exposed mice (0.07 ± 0.0001) and scotopic-exposed mice (0.08 ± 0.003).

### Interaction of Light and Lens Treatment on Dopamine-Related Gene Expression and Proteins

**Figure 6.**
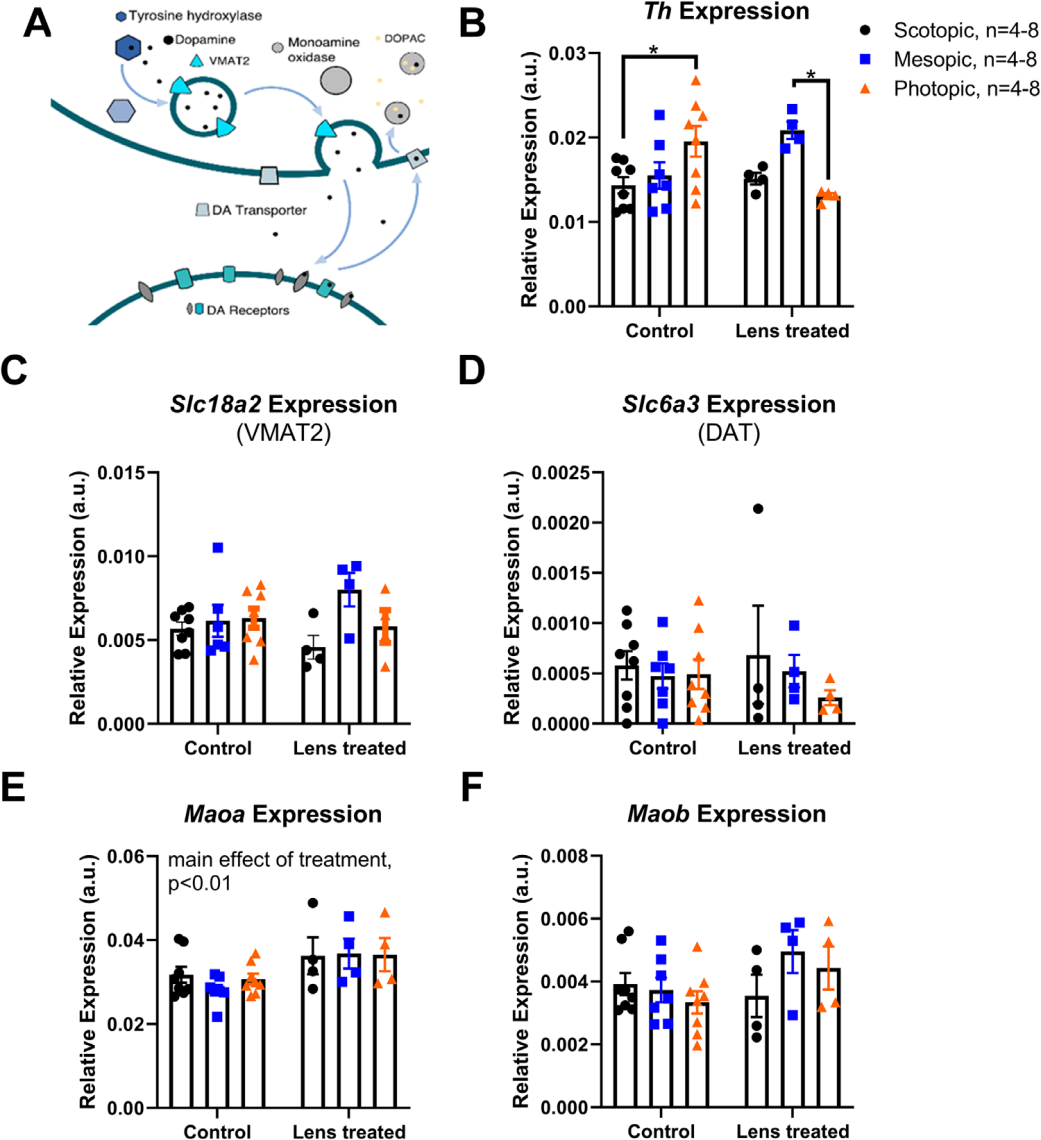
Expression of genes associated with DA signaling. The expression levels of DA signaling genes were measured with ddPCR after light exposure and LIM. (**A**) DA signaling in dopaminergic cells depend on tyrosine hydroxylase and several other related proteins to tightly control levels of DA and its metabolite DOPAC. (**B**) In control mice, *Th* expression was significantly higher in mice housed in photopic light compared to scotopic (two-way ANOVA, interaction effect F(2,29) = 7.52; *P* < 0.01; post hoc comparison; *P* < 0.05). Lens defocus–treated retinas from mesopic light had significantly higher *Th* expression then lens treated, photopic retinas (*P* < 0.05). (**C, D**) No significant differences were found in expression of *Slc6a3* (DAT) or *Slc18a2* (VMAT2). (**E**) LIM eyes were significantly higher than control eyes for expression levels of *Maoa* (two-way ANOVA, main effect of treatment, F(1,29) = 9.92; *P* < 0.01) and (**F**) *Maob* expression did not change with lens or light treatments . Data are mean ± SEM measured in arbitrary units normalized to levels of HPRT. Scotopic samples are represented by *black circles*, mesopic samples by *blue squares*, and photopic samples by *orange triangles*. For post hoc comparisons, *P < 0.05.

Transcripts encoding proteins associated with DA signaling were quantified by ddPCR. *Th* expression was significantly dependent on the interaction of light and lens treatment (two-way ANOVA, interaction effect, F(2,29) = 7.52; *P* < 0.01, Fig. 6B). In control mice, *Th* mRNA levels increased in photopic- compared to scotopic-housed mice (scotopic: 0.014 ± 0.001 arbitrary units (a.u.); photopic: 0.020 ± 0.002 a.u.; *P* < 0.05). In contrast, *Th* expression in lens defocus treated was significantly higher in mesopic-housed mice (0.021 ± 0.001 a.u.) than photopic light–housed mice (0.013 ± 0.000 a.u.; *P* < 0.05). No significant differences in expression of *Slc18a2* or *Slc6a3* were found among treatment groups (Figs. 6C, D). *Maoa* was affected by treatment such that control eyes had lower levels of expression than LIM eyes (two-way ANOVA, main effect of treatment, F(1, 29) = 0.212; *P* < 0.01, Fig. 6E). *Maob* did not change with light or lens treatment (Fig. 6F). Values for contralateral eyes are shown in Supplemental Figure S3.

**Figure 7.**
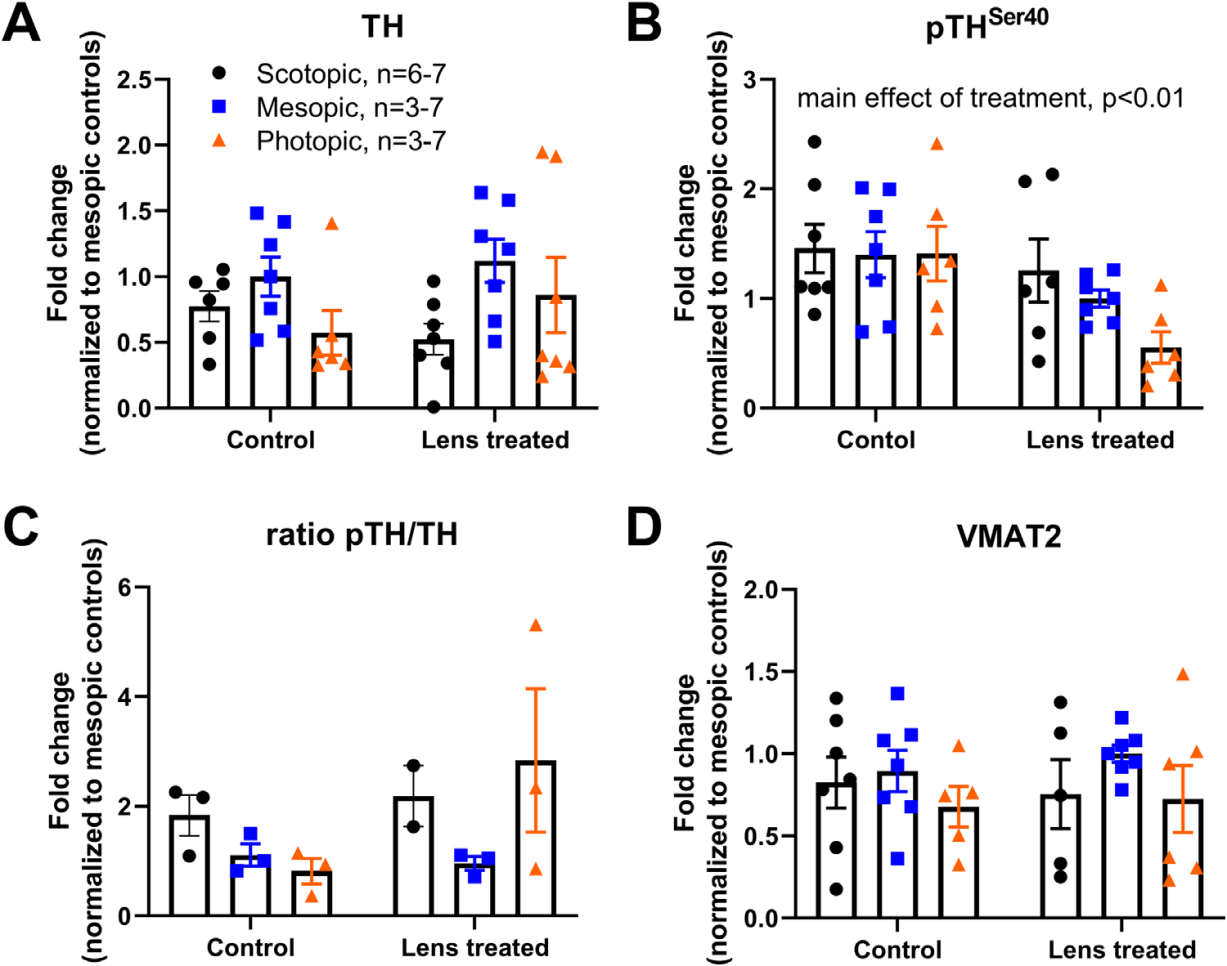
Decreased phosphorylated TH under lens treatment as ambient light increases. (**A**) Levels of TH did not change significantly with ambient light or LIM. (**B**) Phosphorylated TH, pTH^Ser40^, was inversely related to light intensity (two-way ANOVA, main effect of treatment, F(1,33) = 8.34; *P* < 0.01). This effect was likely driven by decreased pTH^Ser40^ levels in lens defocus–treated retinas housed in photopic light (*orange triangles*) compared to mesopic lens–treated retinas (*blue squares*) and scotopic lens–treated retinas (*black circles*) compared to equivalent pTH^Ser40^ in control retinas from all light levels. (**C**) The ratio of pTH^Ser40^ to total TH showed a nonsignificant relationship between control and lens defocused retinas under each light level. Only retinas which were used for TH and pTH^Ser40^ labeling were used in this analysis limiting the sample number. (**D**) VMAT2 protein levels were not significantly affected by light or lens defocus exposure. Data are mean ± SEM. Representative blots are included in Supplementary Figure S4.

No significant differences in tyrosine hydroxylase (TH) protein levels were observed between control and LIM-treated eyes across light levels (Two-way ANOVA [factors: lens, treatment], F(2, 34) = 1.13; *P* = 0.33; Fig. 7A). However, a nonsignificant trend indicated that lens defocus decreased TH levels in retinas exposed to scotopic and photopic light, whereas TH levels may increase in mesopic-exposed retinas, similar to the *Th* gene expression levels (Fig. 6B). Levels of pTH^Ser40^ were stable across light levels in control eyes (1.43 ± 0.02 fold change) but decreased with lens defocus treatment (0.94 ± 0.21 fold change), likely driven by a decrease in LIM-treated retinas with increasing light intensity (two-way ANOVA, main effect of treatment, F(1,33) = 8.34; *P* = 0.007, Fig. 7B). The ratio of phosphorylated to total levels of TH was calculated to determine what portion of TH was actively synthesizing DA under each condition. No differences among groups were found (Fig. 7C). Levels of VMAT2 were also not changed with light or lens defocus treatment (Fig. 7D).

### DA Activity Was Differentially Altered By Acute Light Exposure to Ambient Light Levels

**Figure 8.**
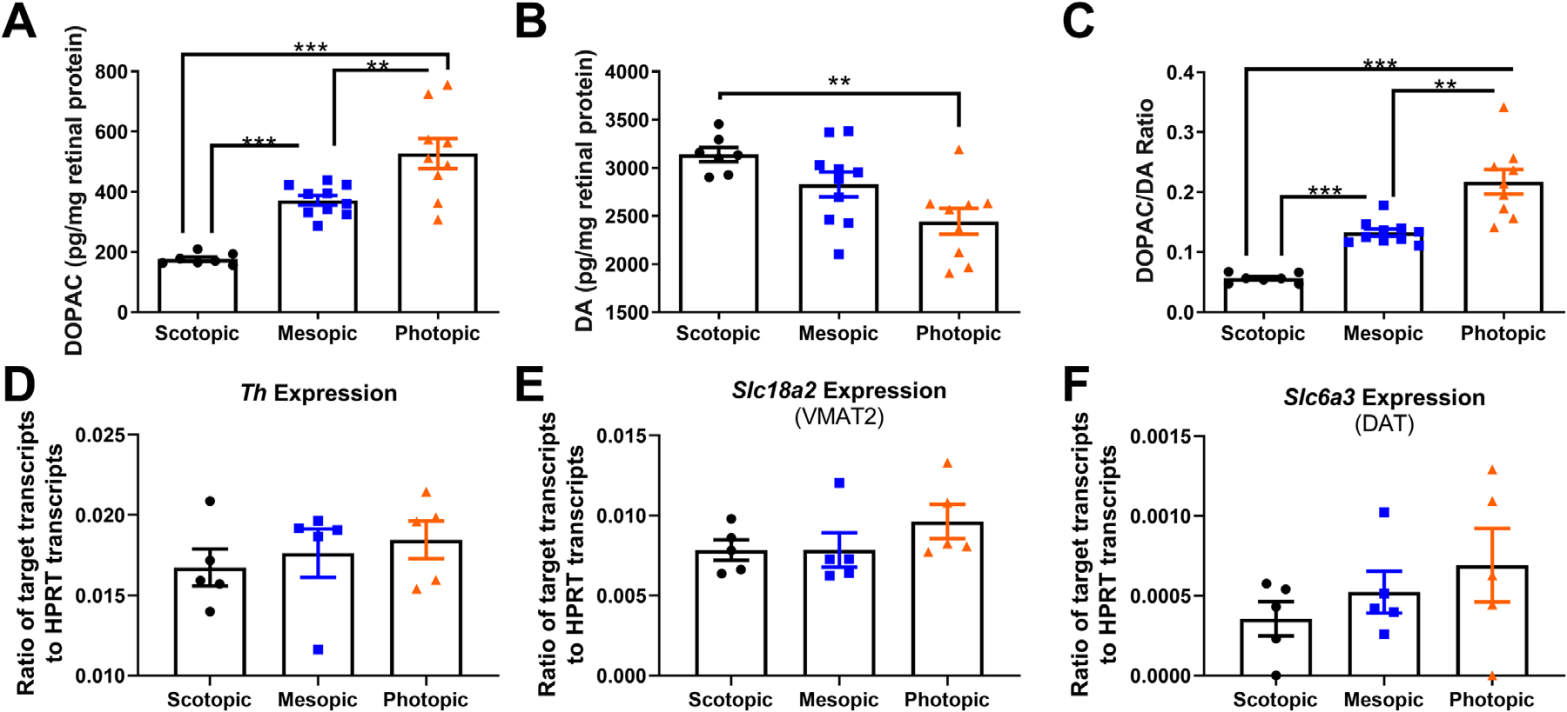
Acute exposure to photopic illuminance drives higher DA activity. (**A**) The levels of DOPAC in retinas exposed to three hours of scotopic (*black circles*, *n* = 5–7), mesopic (*blue squares*, *n* = 5–10), or photopic (*orange triangles*, *n* = 5–9) light indicate an increase in DA metabolism under bright light during short exposures (one-way ANOVA, F(2, 23) = 27.59; *P* < 0.001). (**B**) DA was highest in scotopic light (one-way ANOVA, F(2, 23) = 7.32; *P* < 0.01). (**C**) The DOPAC/DA ratio representing DA activity was lowest in scotopic exposed retinas and highest in photopic light (one-way ANOVA, F(2, 23) = 35.22; *P* < 0.001). The gene expression of (**D**) *Th*, (**E**) *Slc18a2*, (**F**) *Slc6a3* were not significantly different across illuminance level exposures. Data represent as mean ± SEM. Post hoc comparisons are indicated, **P < 0.01, ***P < 0.001.

To test DA adaptation activity after acute (three hours) versus long-term (12:12 for two weeks) ambient exposure to scotopic, mesopic, or photopic light, retinas were collected from mice (n = 7–10/group) and tested using HPLC. Similar to long-term exposure (Fig. 5), levels of DOPAC were highest in photopic light (527.0 ± 49.6 pg/mg, one-way ANOVA, F(2, 23) = 27.59; *P* < 0.001, Fig. 8A) compared to mesopic (371.7 ± 15.9 pg/mg; *P* < 0.001) and scotopic (176.4 ± 7.1 pg/mg; *P* < 0.001). Although DA levels were stable after long-term light exposure, DA levels after acute exposure were higher in scotopic light than in photopic light (scotopic: 3138 ± 73.5 pg/mg; photopic: 2443 ± 134.3 pg/mg; one-way ANOVA, F(2, 23) = 7.32; *P* < 0.01, Fig. 8B). Additionally, DOPAC/DA ratios increased with exposure to higher illuminance (photopic: 0.22 ± 0.02; mesopic: 0.13 ± 0.01; scotopic: 0.06 ± 0.003; one-way ANOVA, F(2, 23) = 35.22; *P* < 0.001; Fig. 8C).

To determine the role of DA-related proteins in this short-term adaptation process, ddPCR was used to test retinas from control mice exposed to the same conditions of three hours of each light level (n = 5/group). No significant differences between expression levels of *Th*, *Slc18a2* (VMAT2), and *Slc6a3* (DAT) were found across any light level (Figs. 8D–8F). However, nonsignificant trends suggested that expression levels are higher in retinas exposed to higher intensity light.

## Discussion

### Exposure to Mesopic Light Increased Myopia Susceptibility Compared to Scotopic and Photopic Light

Using the mouse model of lens defocus myopia, we have shown the importance of a wide range of ambient illuminance levels on myopia development. Scotopic light, like photopic light, attenuated the effects of lens defocus on myopic eye growth while mesopic light increased the myopic shift and resulted in a significant correlation with increased axial length. We hypothesize that the mechanisms controlling these differences are likely based on the retinal circuitry activated by different light levels which in turn alter DA activity. Our data support previous findings that DA turnover increases when the retina is exposed to increasing illuminance levels. However, DA-related gene expression and protein levels were differentially influenced by the three ambient light levels when lens defocus was present. In lens defocus eyes exposed to mesopic lighting, DA synthesis and storage was increased. Why this increased DA activity does not result in increased DA levels or protection from LIM is unclear, but it is possible that extra DA is quickly degraded or does not bind to dopamine receptors, resulting in increased eye growth.

### Retinal Signaling Pathway Underlying Scotopic, Mesopic, and Photopic Light Detection

**Figure 9.**
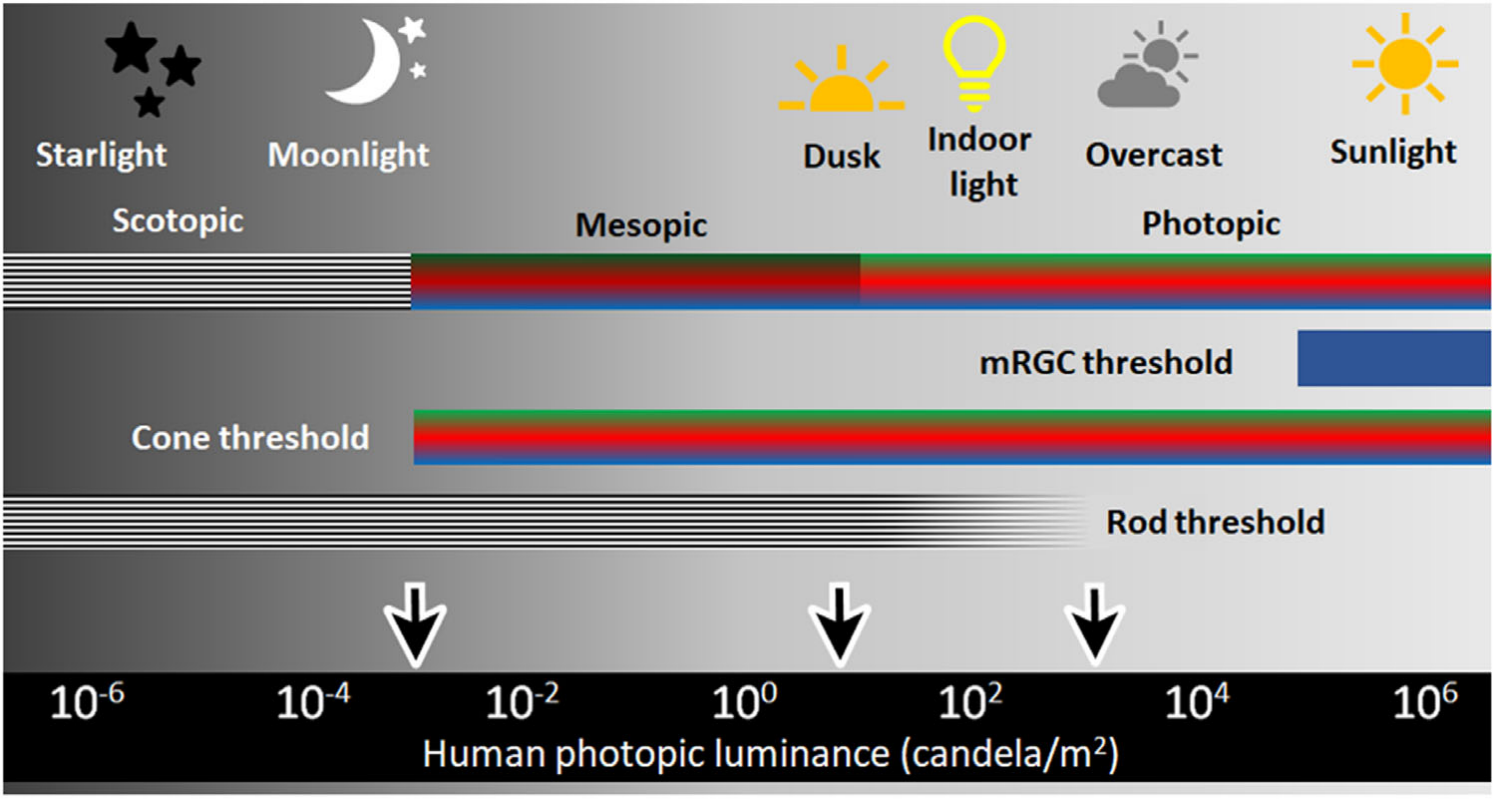
Light sensitivity across photoreceptors. The human photoreceptor sensitivity covers a broad range of natural and artificial light. Scotopic light is below the cone threshold of activation, mesopic light activates both rod and cone photoreceptors, and photopic light activates cone photoreceptors. Recent research has indicated potential roles for melanopsin retinal ganglion cells (mRGCs) and rods in photopic light.^88^^,^^113^ The illuminance levels used in these experiments are indicated with black arrows. Figure is adapted from references 67 and 114 to 116.

For these experiments, we chose specific ambient light levels that would selectively stimulate each photoreceptor type. As shown in Figure 9, scotopic light (1.6 × 10^−3^ cd/m^2^) isolated rod pathways, mesopic light (1.6 × 10^1^ cd/m^2^) activated both rod and cone pathways, and photopic light (4.7 × 10^3^ cd/m^2^) was chosen to stimulate cones. The visual system is able to detect a broad range of light due to the complimentary activation of these various photoreceptor pathways that allow for optimization of vision across drastically different levels of illumination. The photopigments in the photoreceptors have different and complimentary ranges of sensitivity across these light levels (Fig. 8). Additionally, modulation of the inner retina through gap junctions and release of dopamine and nitric oxide either enhance or restrict connectivity between several different cell types in the retina to optimize vision under these different ambient conditions.^49^ Here, we examined how this specialization of the photoreceptors for optimal detection under the three different light levels may contribute to myopia development by carefully selecting ambient illumination based on rod and cone thresholds.

Several studies suggest that rod pathways could play an important role in refractive development. First, hyperopic defocus to the peripheral, rod-dominated retina will induce myopia.^19^ Next, the absence of rod pathway function in the rod transducin knock-out mouse (*Gnat1*−/−) results in abnormal refractive development and absence of a response to form deprivation.^20^ Last, rod photoreceptors synapse onto AII amacrine cells that connect exclusively to ON bipolar cells. ON bipolar cell activation stimulates DA release, a “stop signal” for refractive eye growth.^31^^,^^50^ Furthermore, ON pathways stimulation is also thought to be protective in myopia development because mice with ON pathways defects have increased susceptibility to induced myopia,^36^^,^^51^ and ON pathways stimulation causes choroid thickening in humans and chicks.^52^^–^^54^

An alternative explanation for our results showing that scotopic light is protective for myopia could be that the ambient light levels were too dim to activate the retinal pathways. To confirm that the mice could “see” the scotopic light, we tested the circadian rhythms of mice under each ambient light condition using running wheels and found that mice housed in scotopic light were able to entrain to this light level (Supplementary Fig. S5). Although this data provides some evidence that scotopic light stimulated retinal circuits, it is known that low luminance is correlated with reduced visual acuity^55^^–^^57^ and increased visual blur,^58^^–^^62^ factors that can lead to myopia progression.^62^^–^^66^ Thus one might predict that scotopic light would increase the susceptibility to myopia, instead of decrease it. Further research is needed to determine how dim ambient illumination or dim stimuli can alter retinal processing, particularly the ON and OFF pathways.^60^

Mesopic illuminance range is complex because rod-cone interactions, rod saturation, and mixed photoreceptor spectral sensitivities result in alterations in the spatial properties of the visual system.^67^ In the inner retina, the electrical coupling of AII amacrine cells is low under both starlight (scotopic) and daylight (photopic) conditions, and high under twilight conditions.^49^ The reduced coupling in scotopic and photopic lighting is thought to increase the “fidelity” of the signal in dim light and inhibit lateral interactions for higher acuity in bright light.^49^ Perhaps the mesopic condition here is more similar to the twilight condition in which electrical coupling is high, and this produces conditions where the signaling molecules for eye growth regulation, like dopamine, are not as efficiently released.

Bright ambient light has been protective against induced myopia in animal models and children.^22^ Although cone-driven, high-acuity vision has been hypothesized to underly emmetropization,^68^^–^^72^ the role of cone pathway stimulation in myopia development is unclear. Studies have demonstrated that the cone rich fovea is not essential to induce myopia^18^ and cone-deficient mice do not have altered refractive development, although the susceptibility to form deprivation is increased.^73^

The photopic light chosen here could also stimulate mRGCs. Melanopsin thresholds have typically been defined by pupil light reflex or circadian phase shifting.^74^^,^^75^ However, recent studies suggest that activation of melanopsin may occur in dim light.^76^^,^^77^ A few lines of evidence implicate melanopsin as playing a role in refractive eye growth, including lack of adaptation in melanopsin-driven pupillary response in adult with myopic refractive errors,^78^ identification of altered *Opn4* gene expression in lens-induced myopia in chicken,^79^ altered refractive development and increased response to form deprivation in *Opn4*−/− mice (preliminary data not shown), and mRGC dendrites colocalizing on dopaminergic amacrine cells to potentially modulate DA release.^80^^–^^86^ Alternatively, other studies examining mRGC-driven pupil responses in children found no association with refractive status.^87^ Thus further research is needed to determine whether photopic light might stimulate melanopsin-mediated retinal pathways that slow eye growth.

It is noteworthy that rod photoreceptors have recently been reported to be active under sustained bright light condition.^31^^,^^88^ Thus rod photoreceptor stimulation and rod-mediated DA release could be contributing to the protective effects of scotopic and photopic light on myopia development. In fact, a recent publication suggests that rod photoreceptor activation is solely responsible for the release of dopamine in the retina.^31^ Taken together, these results suggest that ambient light levels could have a differential effect on refractive eye growth through the activation of different retinal pathways.

### Ocular Parameter Changes Associated With Ambient Lighting Conditions

A challenge of using the mouse model of myopia is the small size of the eye. Sensitive techniques are required to detect changes in axial length because schematic eye modeling predicts that only 5 to 6 µm is required for one diopter of refractive change.^89^ Although some studies have reported the expected axial elongation with myopic shifts in mice,^39^^,^^90^^–^^93^ in our hands, this has been difficult to demonstrate.^20^^,^^36^^,^^51^^,^^73^^,^^94^^,^^95^ However, performing correlations between axial length and refractive error reveals clear associations.^96^ In normal mice, combining the data from P28 and P35 creates a positive correlation between axial length and refractive error because of the normal elongation of the mouse eye with age (see Supplemental Fig. S2). However, in eyes with lens defocus, this relationship shifts such that axial length is negatively correlated with refractive error in the mesopic group with the largest myopic shift. The slope of the line is nearly flat in eyes with lens defocus housed in scotopic and photopic, because the myopic shift is present, but small, and thus has less axial elongation.

Corneal curvature flattened with age in the mouse under all ambient lighting conditions. However, under mesopic conditions, lens defocus eyes had significant steepening of the cornea curvature compared to the contralateral or control eyes. This finding may contribute to the increased myopic shift found in mice exposed to mesopic versus scotopic and photopic lighting.

### DA Signaling in the Mouse Model of Myopia

DA has previously been implicated as an antimyopigenic signal in refractive development.^23^^,^^24^^,^^28^ Other studies of DA activity after FDM in mice have not been able to measure change in retinal DA regulation.^20^^,^^95^^,^^97^ Similarly, our analysis of retinal DA and DOPAC levels did not show a difference between lens-treated and control (or contralateral) eyes (Fig. 4). However, there is evidence that DA modulates refractive eye growth in mice: (1) transgenic mice with low retinal DA levels have increased susceptibility to induced myopia,^20^^,^^36^^,^^50^ (2) mice with a retina-specific dopamine deficiency (rTH) have relative myopia compared to wild-type littermates,^29^ (3) reducing DA levels by 6-hydroxydopamine treatment induced myopia shifts^98^ and (4) restoration of DA using L-DOPA treatment prevents FDM.^94^ Collectively, these results support a role for DA in myopic eye growth in mice.

### Interaction Between Illuminance and Lens Defocus Altered DA Signaling

A limitation of many previous studies examining DA and DOPAC in myopia is the analysis of these catecholamines using only HPLC analysis, which provides the total amount of both intracellular and extracellular DA and an estimate of DA metabolism or turnover by measuring the DA metabolite, DOPAC. In the current experiments we examined DA-related gene expression and protein levels and found that the pattern of DA-related activity in the three ambient light conditions was altered by lens defocus. To investigate the multiple steps of DA signaling, we evaluated genes and proteins related to DA synthesis (TH and pTH^Ser40^), storage (VMAT2), uptake (DAT), and degradation (MAO A/B) after both light exposure and lens defocus treatment.

Although control mice showed an increase in *Th* expression with increasing light intensity, lens defocus eyes showed the highest *Th* expression levels in mice exposed to mesopic light. Additionally, there was a trend for TH protein levels to be greatest in mesopic light and lens defocus eyes. Reflecting DA homeostasis, pTH protein levels were stable in the control eyes. With lens defocus, pTH levels decreased with increasing illuminance. Other studies using a single illuminance level reported that *Th* gene expression decreased with myopia,^99^^,^^100^ TH protein levels decreased the response to induced myopia^101^ or TH antibody labeling was unchanged in retinal sections.^97^ One study using both photopic and mesopic light also found an interaction between light level and lens defocus with increased pTH in eyes exposed to bright light.^102^

There was a trend for VMAT2 gene expression and protein levels to be increased in lens defocus eyes exposed to mesopic light, suggesting greater DA storage.^103^ Lens defocus resulted in more DA degradation (*Maoa* expression) across all light levels. Surprisingly, levels of pTH^Ser40^ were significantly decreased in lens-treated eyes with increasing light intensity. These data show an interaction between the lens defocus and the response to ambient light intensity, which together modify DA activity.

Furthermore, the pattern of protection for myopia across light levels used here closely resembles the pattern of DA-mediated cell-to-cell coupling across similar light levels. Coupling between AII amacrine cells is highest in mesopic light and lowest in both scotopic and photopic light.^49^ The inhibition of this coupling is mediated by the release of DA from DACs acting on D1-like receptors found on AII amacrine cells.^104^^–^^106^ Different DA receptor activities, modulated by retinal pathways and DA availability, optimize vision under different lighting conditions and may alter refractive development under long-term or consistent exposure to specific illuminance levels.^107^

Further studies are needed on the potential mechanism of DA signaling in both scotopic and photopic conditions to more fully understand the retinal pathways that are being activated. Further studies would also indicate whether other neurotransmitters, neuromodulators, or transcription factors, including nitric oxide, acetylcholine, GABA, and Egr-1 play a role in these mechanisms.^97^^,^^108^^–^^110^ GABA, like DA, increased with light in these experiments, indicating its potential importance in these mechanisms (Supplementary Fig. S6). These neuromodulators/neurotransmitters have been shown to be related to DA signaling, suggesting that the potential influence of DA on refractive eye growth could be more complex than generally thought.

### DA Homeostasis Under Short and Long-Term Light Exposure

It is well established that the synthesis of retinal DA increases with light onset^30^ and DA activity is stimulated by increasing light intensity.^31^^,^^111^ DOPAC levels and DOPAC/DA ratios were highest in mice housed under photopic light and lowest in mice under scotopic light, as expected, suggesting that with long-term exposure DA release and consequent degradation increased in response to bright light levels. Here, DOPAC levels were not dependent on lens defocus treatment (Fig. 5), similar to previous studies in which DA activity does not seem to change in response to FDM in mice.^20^^,^^94^^,^^95^^,^^97^

In mice with acute exposure, retinas from the photopic light group had the lowest levels of DA and the highest levels of DOPAC. In contrast, retinas from the scotopic light group had the highest DA levels and lowest DOPAC. These results suggest that DA metabolism increases immediately after bright light exposure, but that DA synthesis does not, creating a lag in the overall DA levels. Levels of DA and DOPAC after long-term light housing shows an adaption to bright light such that DA synthesis and metabolism reach homeostasis (Fig. 5B).

In lens defocus experiments, we housed mice in the three different light levels for five days before application of lens defocus to allow for the endogenous retinal DA system to adapt to each light level. This exposure alone did not alter the refractive error but likely altered the level of DA signaling that was occurring at the time of goggling. Further studies are needed to examine how these DA activity changes with adaption to various ambient illuminances could alter later responses to LIM and whether the “preconditioning” of retinal DA levels is needed for the protective effects of scotopic and photopic lighting on lens defocus in mice.

### Clinical Implications

These results suggest that a broad range of ambient lighting conditions in the environment may differentially alter myopia development. Specifically, the time spent in mesopic or indoor ambient lighting conditions could increase the risk or progression of myopia. In support of this, myopic children wearing light sensors to monitor light levels were shown to spend less time in scotopic and photopic light compared to nonmyopic children.^21^ The time myopic children spent in either scotopic or photopic light was approximately equivalent (∼two hours per day).

It is likely that a broad range of ambient light exposure during development, including both dim and bright light, is necessary for healthy ocular growth. Increasing our understanding of the mechanisms of refractive eye growth and myopia development will allow for the development of more efficacious treatments to halt or slow the progression of myopia.

## Supplementary Material

Supplement 1
