# Supplementary material for: Ambient Light Regulates Retinal Dopamine Signaling and Myopia Susceptibility
Source: Invest Ophthalmol Vis Sci. 2021 Jan 27;62(1):28. doi: 10.1167/iovs.62.1.28 (PMC7846952; doi:10.1167/iovs.62.1.28)
Supplement: Supplement 1 [file iovs-62-1-28_s001.pdf]

**Ambient light regulates retinal dopamine signaling and myopia susceptibility**

**Supplemental Information**

**Landis, E., et al.**

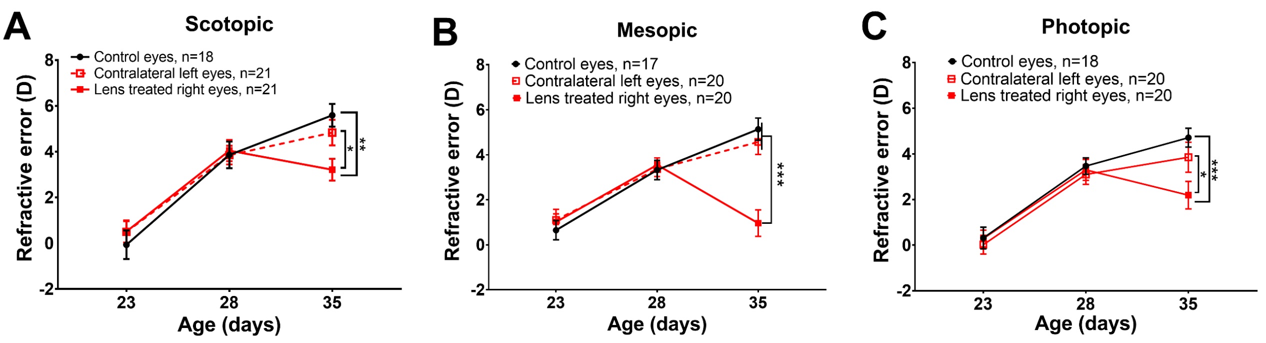


**Supplementary Figure 1. Altered refractive development in lens defocus treated but not contralateral or control eyes.** (A) Mice in scotopic light generally became hyperopic with development. After lens defocus at P28 the lens treated eyes (*solid red*) became relatively myopic compared to their contralateral eyes (*dashed red*, $p<0.05$) and control fellows [*black*, $p<0.01$, RM Two-way ANOVA interaction effect $F(4,114)=5.26$, $p<0.001$]. (B) Mesopic exposed mice have a similar but more severe response to lens defocus. Lens treated mice exhibit greater myopia [RM Two-way ANOVA interaction effect $F(4,108)=13.6$, $p<0.001$] than both contralateral ($p<0.001$) and control eyes ($p<0.001$). (C) Mice in photopic light also responded to lens defocus with a slight myopic refractive error relative to contralateral ($p<0.05$) and control eyes [$p<0.001$, RM Two-way ANOVA interaction effect $F(4,106)=4.49$, $p<0.01$]. All data shown is mean ± SEM, n=17-21/group, post-hoc comparisons indicated by *$p<0.05$, **$p<0.01$, ***$p<0.001$.

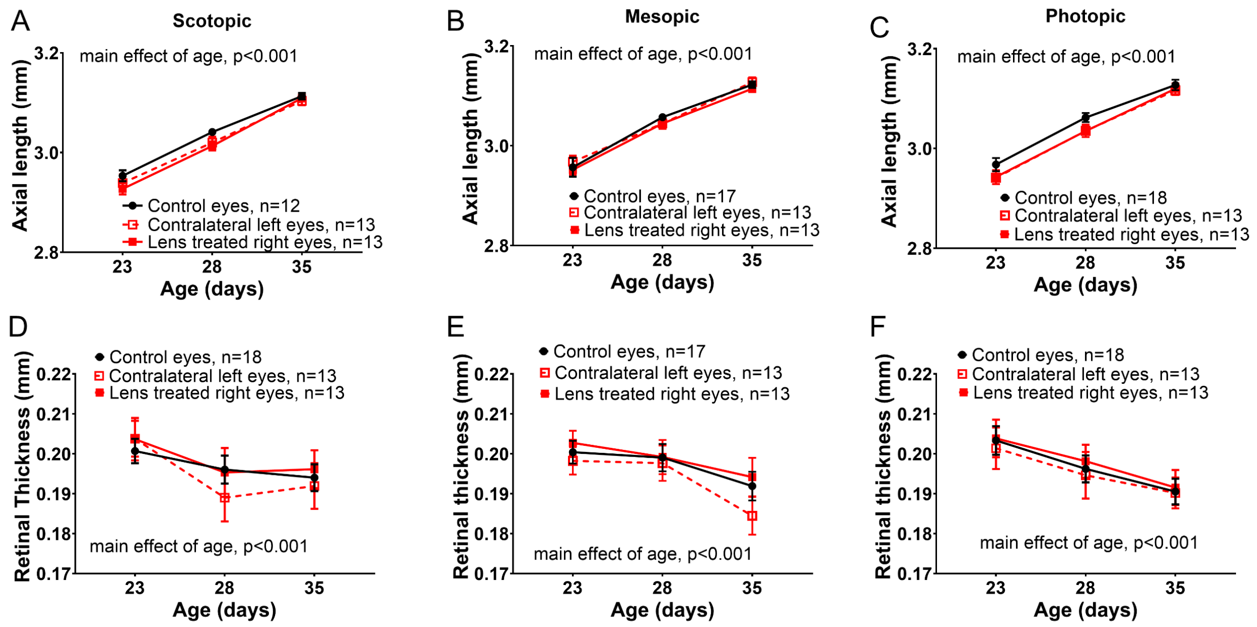


**Supplementary Figure 2. Additional ocular parameters.** Axial length and retinal thickness for mice in scotopic (A, D), mesopic (B, E), and photopic (C, F) light all change with age regardless of lens treatment. Control eyes are shown in *solid black* lines, the naïve left eyes are shown in *red dashed* lines, and lens treated eyes are shown in *solid red* lines. All data shown is mean ± SEM, n=18-21/group.

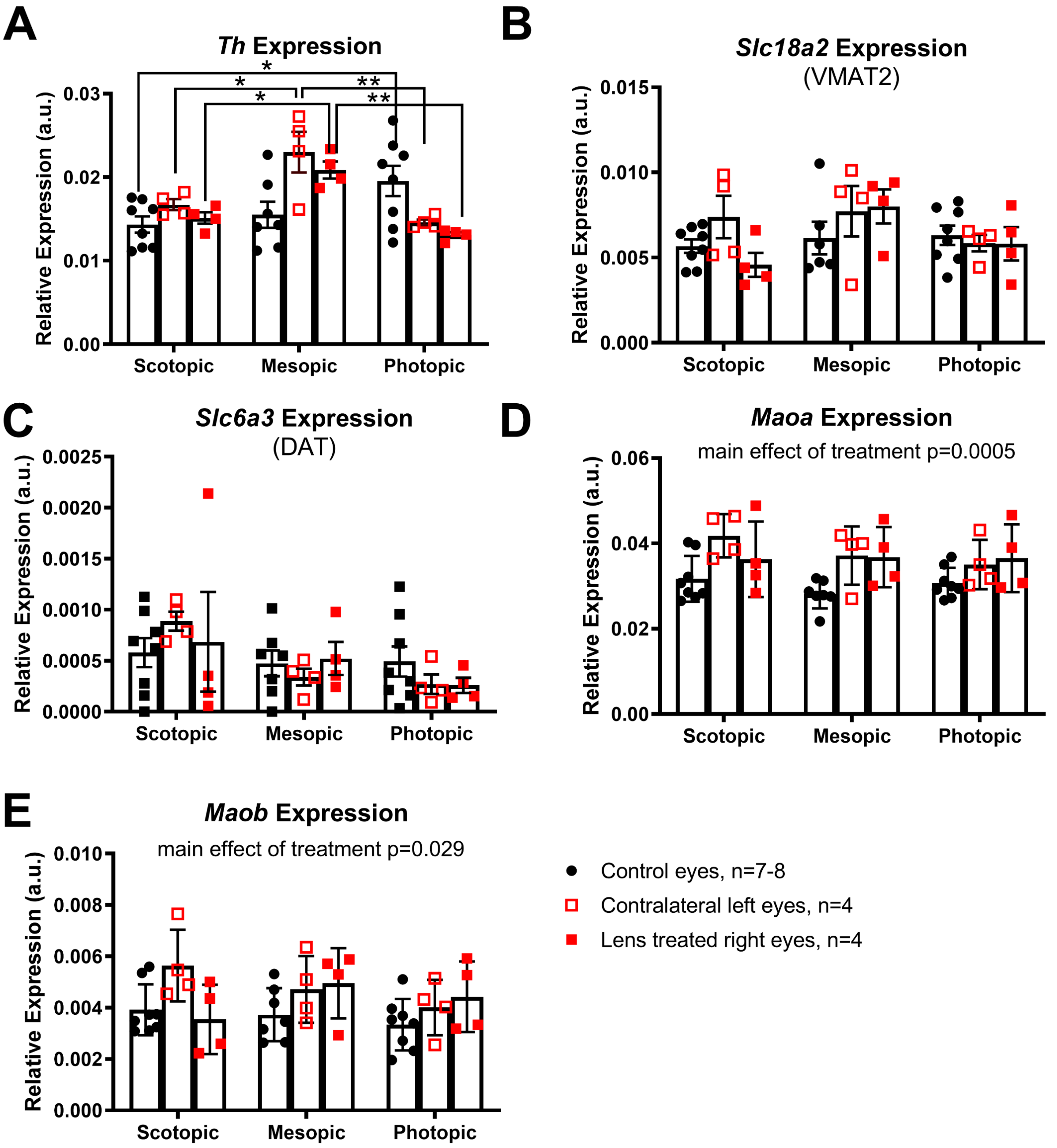


**Supplemental Figure 3: Gene expression associated with DA signaling for the control, contralateral left, and lens treated right eyes**. (A) In control mice, *Th* expression was significantly higher in mice housed in photopic light compared to scotopic [Two-way ANOVA, Interaction effect: $F(4,38)=6.32$, $p=0.005$, Post-hoc comparison, $p=0.012$]. Lens defocus treated and contralateral retinas from mesopic light had significantly higher *Th* expression then lens treated or contralateral retinas from photopic or scotopic light ($p<0.05$). (B-E) No significant differences were found in expression of *Slc6a3* (DAT), *Slc18a2* (VMAT2), *Maoa,* or *Maob.* Data are mean ± SEM measured in arbitrary units normalized to levels of HPRT. For post-hoc comparisons, *$p<0.05$, **$p<0.01$. Data also shown in Figure 5.

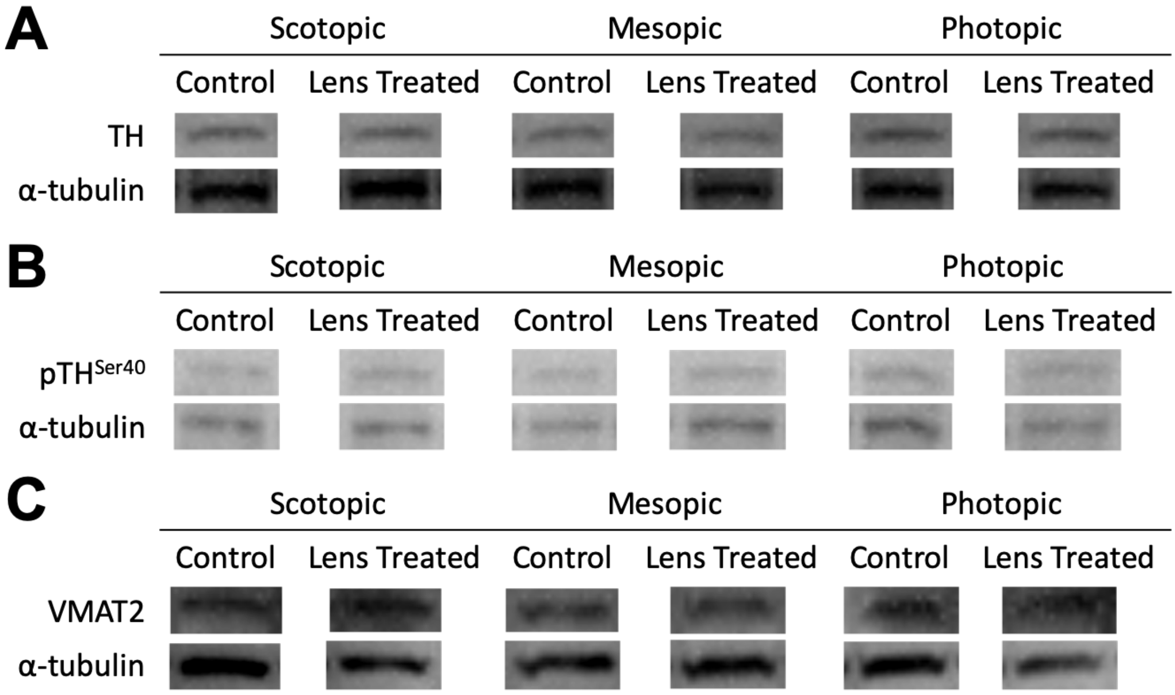


**Supplementary Figure 4. Representative western blots of target DA signaling proteins.** To measure changes to DA-related protein content in retinas exposed western blots were done for (A) TH, (B) VMAT2, and (C) $pTH^{Ser40}$. To confirm proper protein loading alpha-tubulin was labeled as a control.

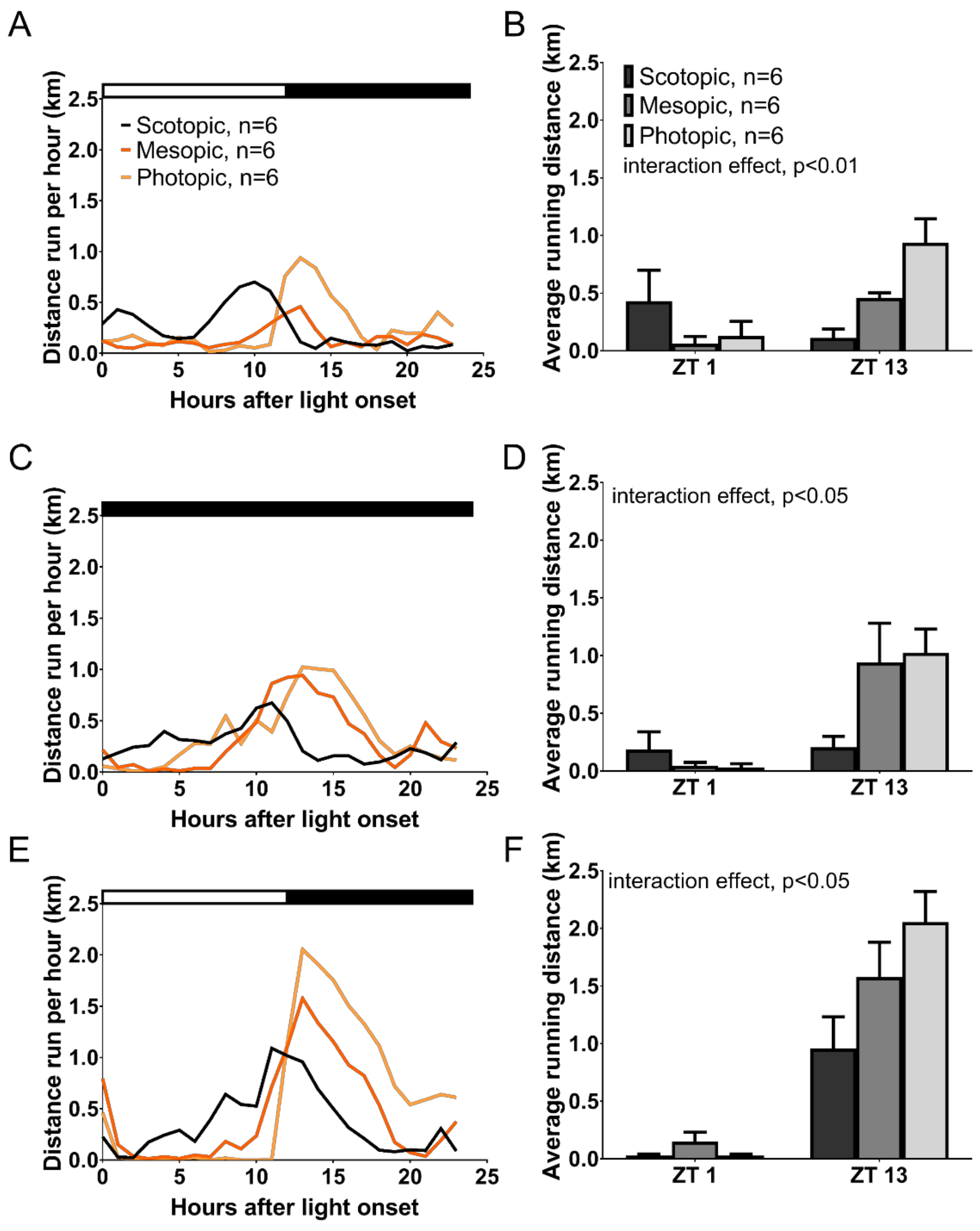


**Supplementary Figure 5. C57BL/6J mice maintained circadian rhythms under all three experimental illuminance levels.** To ensure animals could detect and entrain to the scotopic light, running wheel activity was measured from six animals in each lighting condition to track circadian rhythms. Running distances were calculated by the software based on the number of wheel rotations counted (Med Associates, Inc; Wheel Analysis Software, Fairfax, VT). (A) During the first week, under a normal 12-hour LD cycle, mice in all light levels showed a consistent pattern of running activity that fluctuated with light. (B) Analysis of average running distance at ZT 1 and ZT 13, one hour after light onset and one hour in to the dark phase, respectively, showed a main effect of time such that mice in photopic and mesopic light ran significantly less at ZT 1 than at ZT 13 [Two-way ANOVA, $F(1,35)=5.54$, main effect of time, $p<0.05$]. (C) During the following week of constant darkness, mice maintained running activity peaks during the subjective dark phase but began to increase running during subjective day. (D) All mice ran less at circadian time 1 (CT 1) than CT 13 resulting in a main effect of time [Two-way ANOVA, $F(1,35)=18.87$, main effect of time, $p<0.001$]. (E) For the third week, the 12-hour light cycle was restored. Mice in all light levels responded by re-entraining to the light. (F) With the light cycle resumed to test entrainment, all mice ran significantly more in the dark than in the light [Two-way ANOVA, $F(1,35)=63.35$, main effect of time, $p<0.001$]. Overall, C57BL/6J mice showed the ability to entrain to scotopic, mesopic, and photopic light levels used here.

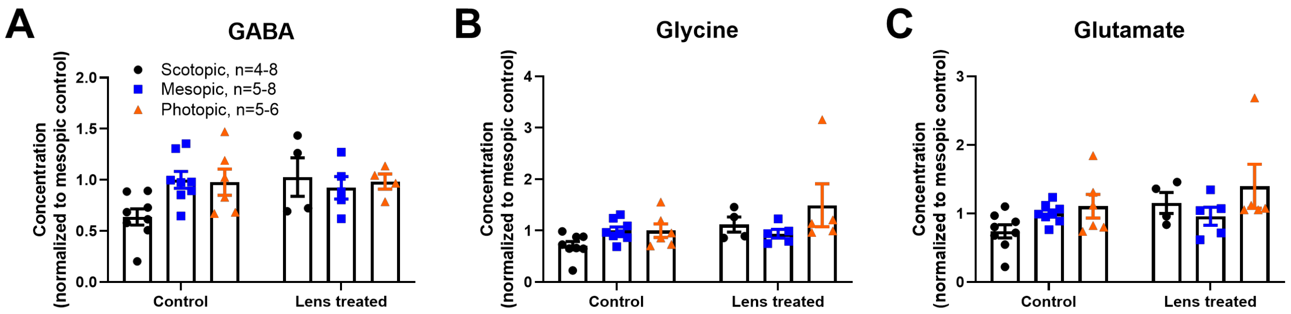


**Supplementary Figure 6. Inhibitory and excitatory amino acid neurotransmitters not altered by light levels or lens defocus.** Using high performance liquid chromatography (HPLC), neurotransmitters were measured from retinas of mice housed in each of the three light conditions. 0.1M perchloric acid was added to homogenized samples which were then filtered to remove debris. Supernatant was injected into an ESA 5600A CoulArray detection system. Separations were performed at 28-30 °C using an MD-150 × 3.2 mm C18 column. The mobile phase consisted of 1.4-1.7 mM 1-octanesulfonic acid sodium, 75 mM NaH2PO4, 0.025% triethylamine, and 8% acetonitrile at pH 2.93-3.0. Analytes were identified by retention time relative to known standards (Sigma Chemical Co., St. Louis, MO) and quantified based on peak area. (A) GABA was measured from control, contralateral, and lens treated retinas. No differences were found with light levels or lens defocus. (B, C) Glycine and glutamate did not change with light or lens treatment. Scotopic samples are represented by *black circles*, mesopic samples by *blue squares*, and photopic samples by *orange triangles*. Data represent mean ± SEM.
